# Translocation of Adenosine A2B Receptor to Mitochondria Influences Cytochrome P450 2E1 Activity after Acetaminophen Overdose

**DOI:** 10.3390/livers4010002

**Published:** 2023-12-26

**Authors:** Giselle Sanchez-Guerrero, David S. Umbaugh, Abhay A. Ramachandran, Antonio Artigues, Hartmut Jaeschke, Anup Ramachandran

**Affiliations:** 1 Department of Pharmacology, Toxicology and Therapeutics, University of Kansas Medical Center, 3901 Rainbow Blvd, MS 1018, Kansas City, KS 66160, USA; 2 Department of Biochemistry, University of Kansas Medical Center, 3901 Rainbow Blvd, MS 1018, Kansas City, KS 66160, USA

**Keywords:** acetaminophen, mitochondria, outer membrane, A2BAR, PGRMC1, Cyp2E1

## Abstract

The adenosine A2B receptor (A2BAR) is a member of a family of G-protein coupled receptors (GPCRs), which has a low affinity for adenosine and is now implicated in several pathophysiological conditions. We have demonstrated the beneficial effects of A2BAR activation in enhancing recovery after acute liver injury induced by an acetaminophen (APAP) overdose. While receptor trafficking within the cell is recognized to play a role in GPCR signaling, its role in the mediation of A2BAR effects in the context of APAP-induced liver injury is not well understood. This was investigated here, where C57BL/6J mice were subjected to an APAP overdose (300 mg/kg), and the temporal course of A2BAR intracellular localization was examined. The impact of A2BAR activation or inhibition on trafficking was examined by utilizing the A2BAR agonist BAY 60–6583 or antagonist PSB 603. The modulation of A2BAR trafficking via APAP-induced cell signaling was explored by using 4-methylpyrazole (4MP), an inhibitor of Cyp2E1 and JNK activation. Our results indicate that APAP overdose induced the translocation of A2BAR to mitochondria, which was prevented via 4MP treatment. Furthermore, we demonstrated that A2BAR is localized on the mitochondrial outer membrane and interacts with progesterone receptor membrane component 1 (PGRMC1). While the activation of A2BAR enhanced mitochondrial localization, its inhibition decreased PGRMC1 mitochondria levels and blunted mitochondrial Cyp2E1 activity. Thus, our data reveal a hitherto unrecognized consequence of A2BAR trafficking to mitochondria and its interaction with PGRMC1, which regulates mitochondrial Cyp2E1 activity and modulates APAP-induced liver injury.

## 1. Introduction

Adenosine receptors are a family of G-protein coupled receptors (GPCRs) that have traditionally been studied due to their mediation of the pathophysiological effects of the purine nucleoside, adenosine. These receptors include the A1, A2A, A2B, and A3 adenosine receptors (ARs) [1]. Among these, the A2BAR is classified as a low-affinity adenosine receptor, which is only activated by micromolar levels of adenosine [2]. Cellular signaling by A2BAR involves the activation of adenylyl cyclase, leading to increased intracellular cAMP levels and the activation of protein kinases [3], which mediate downstream events. While canonical A2BAR activation occurs through receptor activation on the cell surface, it is recognized that receptor trafficking is also an important component of receptor regulation, having an impact on receptor desensitization and cell signaling [4,5]. Receptor localization seems to be cell type- and stimulus-specific, since A2BAR has been shown to be intracellular at rest in intestinal epithelial cells, with apical or basolateral A2BAR stimulation resulting in the recruitment of A2BAR to the plasma membrane [6]. However, it has also been shown that A2BAR can localize to mitochondria in cardiac myocytes [7], with the deletion of A2BAR suppressing the expression of genes involved in mitochondrial biogenesis and function, as well as the downregulation of genes involved in oxidative metabolism in skeletal smooth muscle [8].

Acetaminophen overdose is the most common cause of drug-induced hepatotoxicity and acute liver failure (ALF) in the United States. Liver toxicity after an APAP overdose is due to the excessive cytochrome P450 (CYP)-mediated formation of a reactive metabolite, N-acetyl-p-benzoquinone imine (NAPQI), which depletes hepatic glutathione stores and subsequently induces mitochondrial dysfunction and hepatocyte necrosis [9]. CYP2E1 is the CYP family member predominantly implicated in NAPQI formation from APAP [10,11], and while it is predominantly localized in the ER, mitochondrial CYP2E1, which requires mitochondrial-specific electron transfer proteins for activity, has also been identified in the liver [12]. Hepatic CYP2E1 has been shown to be stabilized by the heme-binding protein progesterone receptor membrane component 1 (PGRMC1) [13], which has also been localized to mitochondria [14]. Notably, studies involving PGRMC1 knockout mice have demonstrated reduced cytochrome P450 activity, with resultant protection against APAP-induced liver injury [13]. We earlier demonstrated that the activation of A2BAR by its agonist BAY 60–6583, when administered beyond the injury phase, promoted the reparative anti-inflammatory immune response and facilitated liver recovery after an APAP overdose [15,16]. However, the influence of A2BAR intracellular trafficking in mediating its hepatic effects is not well understood, and our aim was to characterize A2BAR trafficking in the liver after exposure to an APAP overdose. Thus, the present study focused on changes in A2BAR subcellular compartmentalization over time and the consequences of A2BAR trafficking in the context of APAP pathophysiology. We demonstrate that A2BAR is translocated to the outer mitochondrial membrane after an APAP overdose, binding to PGRMC1 and influencing mitochondrial Cyp2E1 activity.

## 2. Materials and Methods

### 2.1. Animals and Experimental Design

Eight- to ten-week-old male C57BL/6J mice with an average weight of 20–25 g were purchased from Jackson Laboratories (Harbor, ME, USA). All animals were kept in an environmentally controlled room with a 12 h light/dark cycle and free access to food and water. All experimental protocols were approved by the Institutional Animal Care and Use Committee of the University of Kansas Medical Center and followed the criteria of the National Research Council for the care and use of laboratory animals. Mice were fasted overnight (16 h), intraperitoneally (ip) injected with 300 mg/kg of APAP (dissolved in saline), and euthanized under isoflurane anesthesia at 3, 6, and 24 h to evaluate A2BAR translocation. Animals were re-fed at 6 h for the 24 h group. For experiments using 4-methylpyrazole (4MP), animals were treated with 50 mg/kg of 4MP [17] (dissolved in saline, i.p) 1.5 or 3 h after APAP overdose and euthanized at 3 or 24 h after APAP overdose, respectively. For experiments with BAY 60–6853, mice were treated with APAP, followed by BAY 60–6843 [16] (4 mg/kg in DMSO, i.p) or DMSO alone (APAP + DMSO group) 6 h after APAP overdose and sacrificed at 24 h. Some animals were also treated with PSB 603 [18,19], an A2BAR inhibitor (1 mg/kg in 50% DMSO, i.p), at 6 h after APAP overdose and sacrificed at 24 h. Blood was collected from the vena cava using a heparinized syringe and centrifuged at 18,000× *g* for 3 min at 4 °C to collect plasma. The liver was resected and either snap-frozen in liquid nitrogen and stored at *−*80 °C, fixed in 10% paraformaldehyde for histology, or used for subcellular fractionation.

### 2.2. Biochemical Measurements, Histology, and Cyp2E1 Activity Assay

Plasma alanine aminotransferase (ALT) activities were measured with the Point Scientific ALT test kit (Point Scientific Inc., Canton, MI, USA) per the manufacturer’s instructions. Liver protein was measured using the BCA assay (Pierce Scientific, Waltham, MA, USA). For histology, liver tissue was fixed and embedded in paraffin, and 5 μm sections were generated. These were stained with hematoxylin and eosin (H&E) for the assessment of liver necrosis. Areas of necrosis were determined using ImageJ software (version 2.14.0/1.54f) by scanning the entire tissue section for 3 to 5 mice in each group and calculating the percentage of the necrotic area for each liver section. Cyp2E1 enzyme activity was assessed in liver mitochondrial fractions using a fluorogenic substrate, 7-ethoxy-4-trifluoromethyl coumarin (7-EFC; Invitrogen, Carlsbad, CA, USA), as described [20].

### 2.3. Mitochondria and Plasma Membrane Separation

For subcellular fractionation, the right and caudate lobes of the liver were minced and homogenized in ice-cold isolation buffer (at pH 7.4, containing 22 mM mannitol; 70 mM sucrose; 2.5 mM 4-(2-hydroxyethyl)-1-piperazineethanesulfonic acid (HEPES); 10 mM EDTA; 1 mM ethylene glycol tetraacetic acid; 0.1% bovine serum albumin; Halt^™^ Phosphatase Inhibitor Cocktail (Cat# 78420, Thermo Fisher Scientific^™^, Waltham, MA USA); Leuptin 1 μg/μL; Aprotinin 1 μg/μL; Pepstatin 0.1 μg/μL) with 35 strokes using a tight-fitting motorized Teflon pestle. Cell debris was removed by spinning the homogenate at 2500 g for 10 min, and the resulting supernatant was then centrifuged at 20,000× *g* for 10 min to pellet mitochondria. The supernatant was preserved as the post-mitochondrial supernatant containing ER. The plasma membrane was isolated using the Minute^™^ Plasma Membrane Protein Isolation and Cell Fractionation Kit (Invent Biotechnologies Inc., Plymouth, MA, USA) in accordance with the manufacturer’s protocol and resuspended in lysis buffer.

### 2.4. Proteinase K Digestion and Western Blotting

Isolated liver mitochondria (500 μg) were resuspended in mitochondrial isolation buffer and incubated with different concentrations of proteinase K (Sigma Aldrich P6556, St. Louis, MO, USA) for 15 min on ice. Digestion was terminated using 0.1 mM Phenylmethane-sulfonyl fluoride (PMSF), followed by further incubation on ice for 15 min. Loading buffer was added, and proteins were denatured at 95 °C for 5 min. Identical volumes (25 μL) were then separated using SDS-PAGE and analyzed via Western blotting, where samples were separated via SDS-Gel electrophoresis, transferred to nitrocellulose membranes, and blocked in 5% milk diluted in buffered saline with tween-20 (TBST) for 1 h. Blots were incubated with primary antibodies overnight at 4 °C, followed by incubation with the respective secondary antibody for 1–2 h. Proteins were then visualized via enhanced chemiluminescence detection (Amersham Biosciences, Inc., Piscataway, NJ, USA) on the LICOR Odyssey Imager (LICOR Biosciences). Densitometry was performed using Image Studio Lite ver. 5.2 or Image J Software. The following primary antibodies were used: A2BAR (#ab229671) and Cyp2E1 (#ab28146) from Abcam; VDAC (#4866), β-actin (#4970), −2 (#89326S) and PGRMC1 (#13856S) from Cell Signaling Technology; Flotillin (Sigma Aldrich #F1180) and Tom 20 (Santa Cruz Biotechnology sc-11415). The secondary antibodies used for detection were HRP-anti-rabbit IgG (G21234, Invitrogen, Carlsbad, CA, USA) and HRP-anti-mouse IgG (Cell Signaling Technology #7076, Danvers, MA, USA).

### 2.5. Molecular Modeling

The protein–protein docking simulations conducted to predict the binding of A2BAR (PDB: 8HDO) and PGRMC1 (PBD: 4X8Y) were performed by using the web server ClusPro version 2.01 [21], with default options. This program performs rigid body docking using PIPER, a correlation approach based on fast Fourier transform (FFT). This model uses extensive sampling (>109 structures), followed by the clustering of a restricted number of conformations (*~*1000) with the most favorable score, and energy minimization to remove steric clashes to yield the final set of selected predictions. The models obtained after docking A2BAR-PGRMC1 were evaluated for their binding free energies and dissociation constants (Kd) using the PRODIGY3 program [22]. The IC value in PRODIGY is defined as the number of interfacial contacts classified as polar, apolar, or charged according to the nature of these residues. Two residues are defined as being in contact if two of their heavy atoms are within 5.5 Å, and the properties of non-interacting surfaces (NIS) are also considered. The best docking poses of A2BAR-PGRMC1 were subsequently refined in molecular simulations under full hydration conditions using PyMOL Molecular Graphics System, Version 2.0 Schrödinger, LLC.

### 2.6. Statistics

All results are expressed as mean ± SEM. We assessed normality through visual inspection using histograms for small sample sizes or by performing a Shapiro–Wilk test. Subsequently, we conducted a *t*-test for pairwise comparisons and a one-way ANOVA for multiple groups, relying on the assumption of normality within each group, followed by post hoc analysis (Tukey–Kramer). A two-tailed alpha was set at 0.05 (*p* < 0.05). Statistical analysis was performed using GraphPad Prism 9.

## 3. Results

### 3.1. APAP Overdose Induces Mitochondrial Translocation of A2BAR

**Figure 1.**
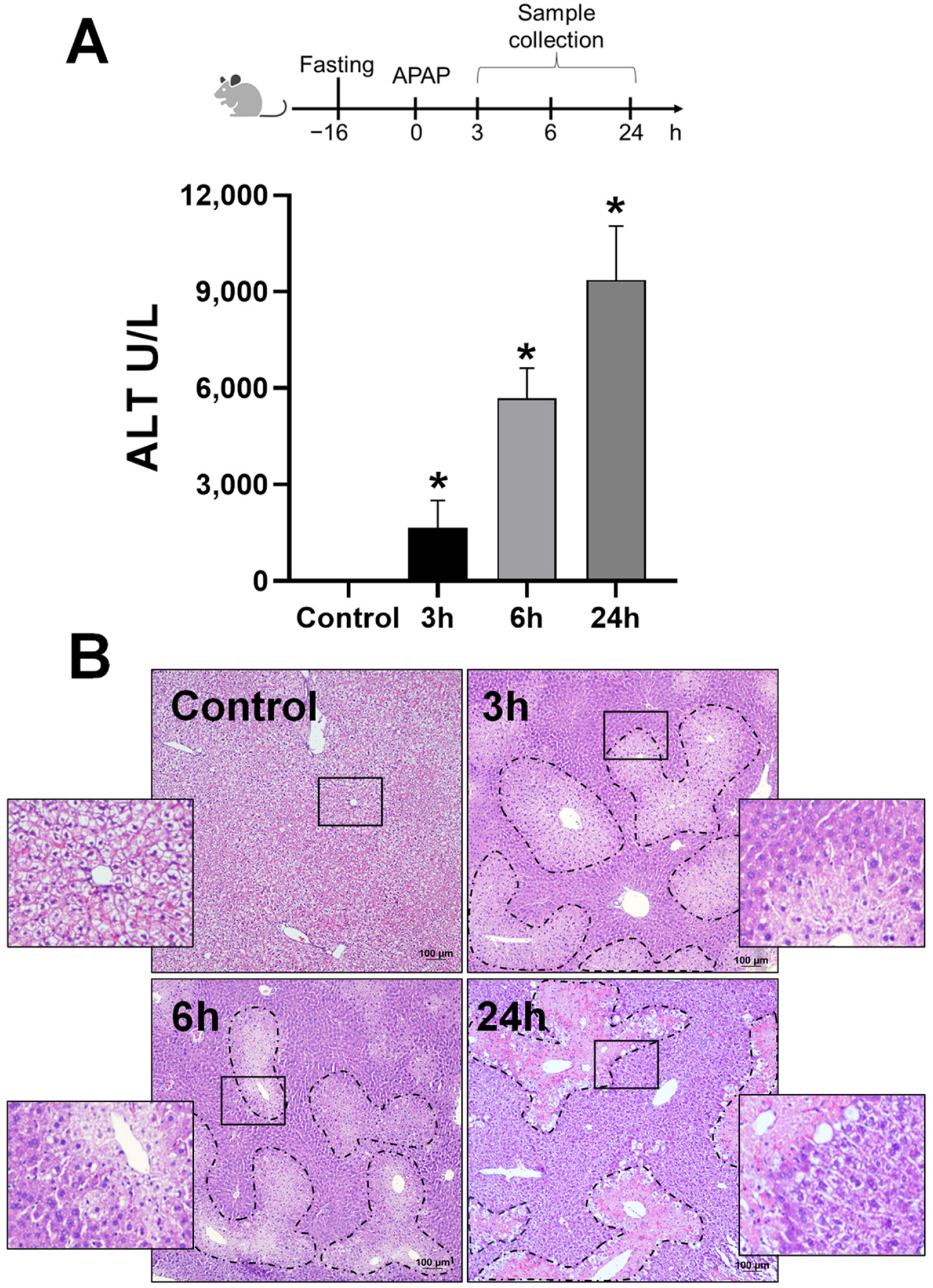
Time course of APAP-induced liver injury. Male C57BL/6J mice were treated with 300 mg/kg of APAP. Blood and liver tissues were obtained at 3, 6, and 24 h post-APAP overdose. (**A**) Plasma alanine aminotransferase (ALT) activity. (**B**) H&E-stained liver sections (10×); dashed lines represent the necrotic area. The inset represents a magnification of marked regions. Data represent means ± SE of *n* = 4–5 animals per group. * *p* < 0.05 (compared to control).

**Figure 2.**
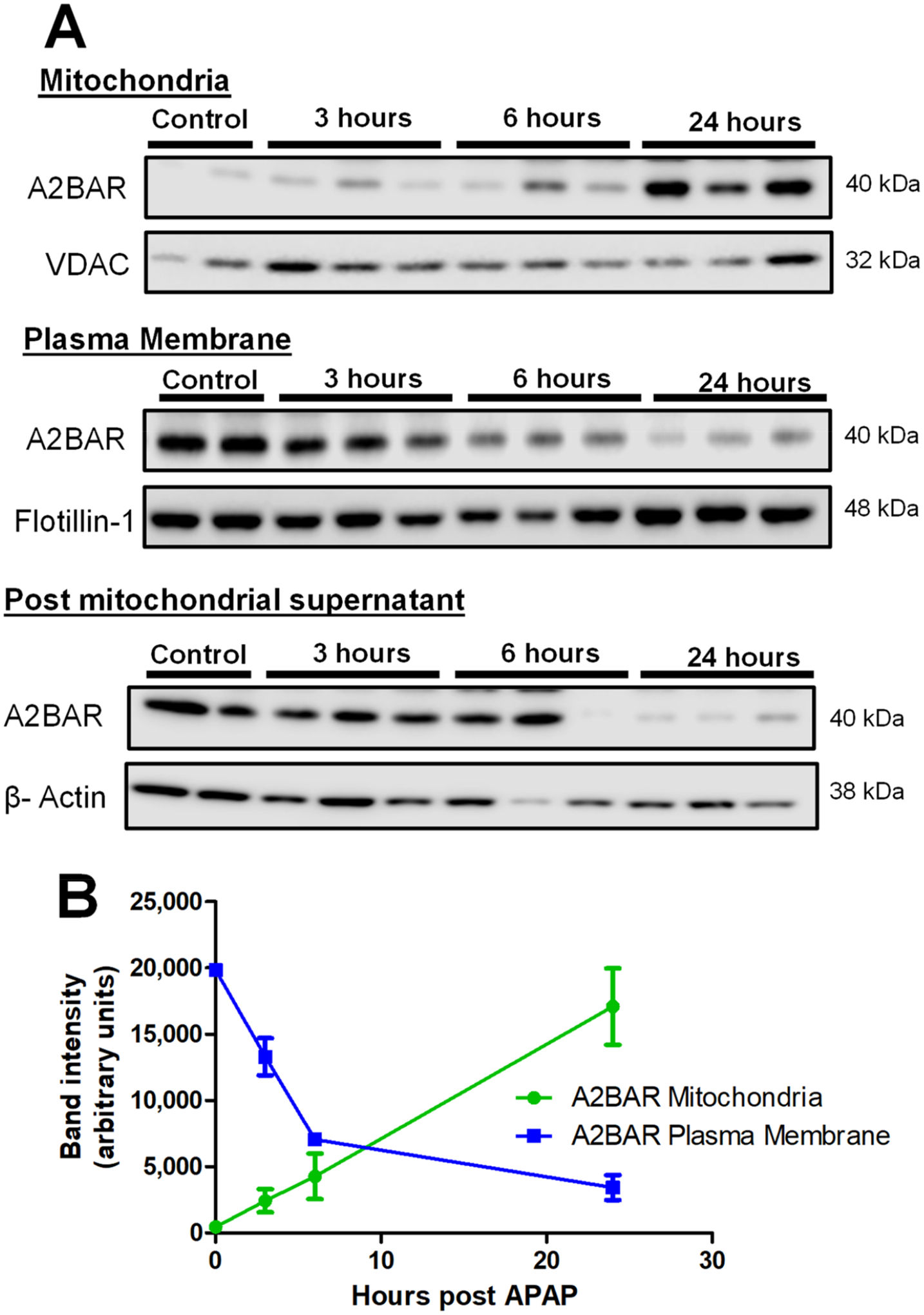
APAP overdose induces A2BAR translocation to the mitochondria. Male C57BL/6J mice were treated with 300 mg/kg of APAP, and liver tissue was collected at 3, 6, and 24 h after APAP overdose followed by subcellular fractionation as described in the methods section. (**A**) A2BAR protein levels in mitochondria, plasma membrane, and post-mitochondrial supernatant. (**B**) Densitometry of A2BAR levels over time in mitochondrial and plasma membrane fractions.

The time course of liver injury after an APAP overdose was initially established, and we examined liver injury after an APAP overdose of 300 mg/kg over the time course of 3, 6, and 24 h by evaluating serum ALT and by conducting a histopathological analysis of liver sections. As expected, treatment with APAP resulted in a significant elevation in ALT levels (Figure 1A) and a progressive increase in centrilobular necrosis during the time course (Figure 1B). Though the adenosine A2B receptor (A2BAR) is a GPCR, previous studies have demonstrated its presence in the mitochondria of cardiomyocytes [23], though its intracellular localization and trafficking in hepatocytes has not been studied. Since mitochondria are central to APAP-induced hepatotoxicity [24,25], we next evaluated relative A2BAR localization in the plasma membrane and mitochondria within the liver along the time course after an APAP overdose. Control animals showed A2BAR localization predominantly in the plasma membrane and post-mitochondrial supernatant (Figure 2A). However, after treatment with APAP, A2BAR demonstrated progressively increasing mitochondrial localization, with mitochondrial levels detectable by 3 h, increasing at 6 h with significant mitochondrial localization by 24 h after APAP (Figure 2A). Interestingly, this increase in mitochondrial A2BAR levels was accompanied by a gradual decrease in A2BAR levels on the plasma membrane (Figure 2A,B) over time. Levels in the post-mitochondrial supernatant, which includes the ER, were also substantially decreased by the 24 h time point. This suggests the trafficking of the A2BAR onto mitochondria from other cellular compartments after APAP overdose.

**Figure 3.**
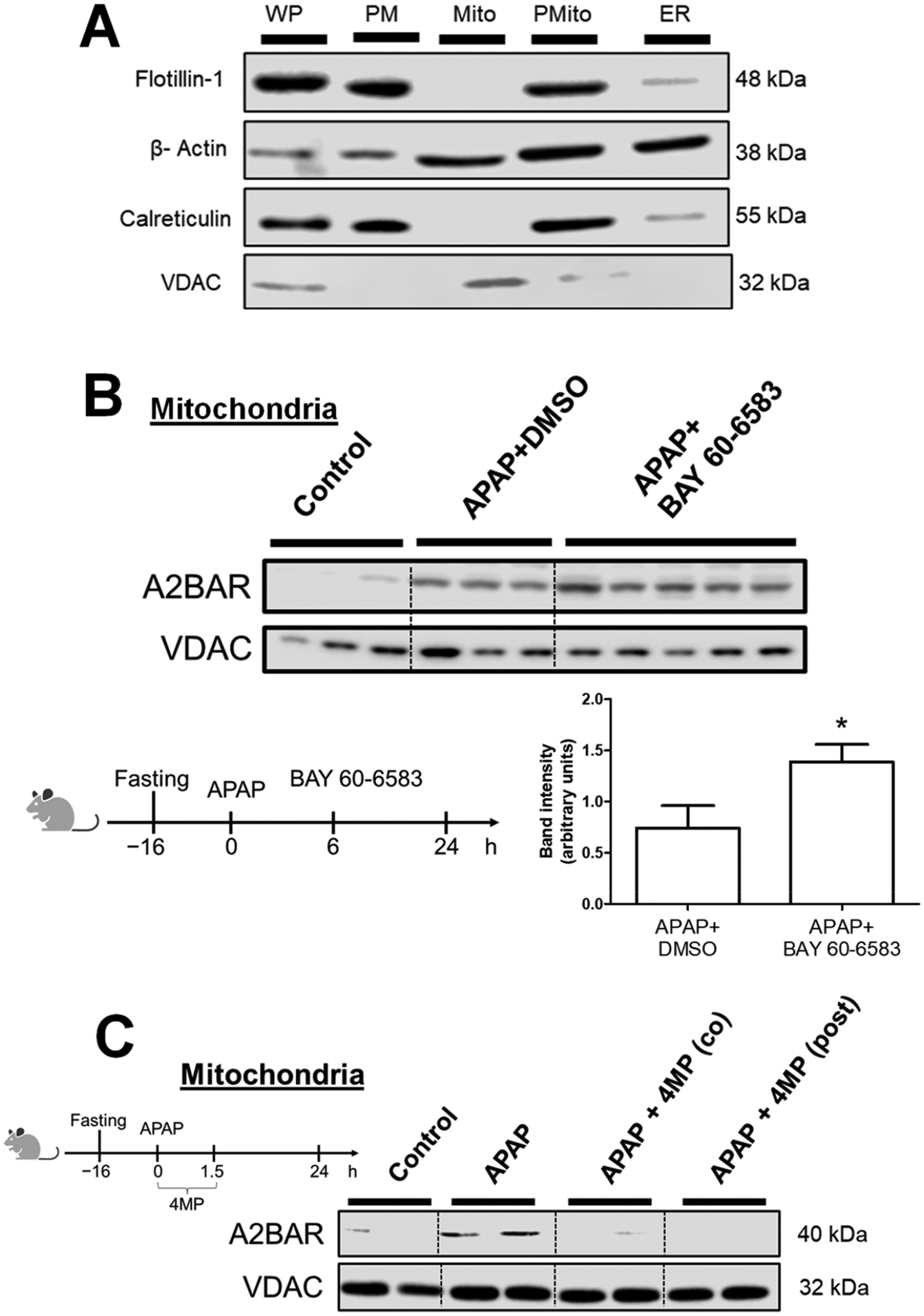
Mitochondrial purity and modulation of A2BAR translocation by its agonists or via the activation of JNK. Mice were administered 300 mg/kg of APAP, along with 4-methylpyrazole (4MP) (50 mg/kg), either as a cotreatment or 90 min after APAP overdose, or BAY 60–6583 (4 mg/kg) 6 h after APAP overdose. (**A**) Western blotting of subcellular fractions against markers for plasma membrane (PM), mitochondria (Mito), post-mitochondrial supernatant (PMito), endoplasmic reticulum (ER), and whole protein lysate (WP) 24 h after APAP overdose. (**B**) Mitochondrial A2BAR determined via Western blotting and densitometry 24 h after APAP overdose with/without treatment with BAY 60–6583 6 h post-APAP overdose. (**C**) A2BAR mitochondrial levels after 4 MP co-treatment or 90 min post-APAP treatment. Data represent means ± SE of n = 3–5 animals per group. * p < 0.05 (compared with APAP + DMSO group)

To establish the purity of mitochondria used in the experiments and confirm that A2BAR mitochondrial localization was not due to a non-specific association of plasma membrane proteins on the organelle after hepatocyte necrosis at the 24 h time point, organelle marker proteins were next examined. As seen in Figure 3A, the plasma membrane protein Flotillin-1 was predominantly present in the plasma membrane fraction and absent in the mitochondria, indicating that mitochondrial A2BAR was not an artifact of hepatocyte cell death. The mitochondrial fraction also did not show significant ER contamination as indicated by the lack of the ER marker calreticulin. The presence of calreticulin on the plasma membrane is not surprising since the protein has been shown to be present on the surface of mammalian cells [26]. Taken together, these data indicate that the mitochondrial presence of A2BAR was due to bonafide translocation over time after APAP overdose and not an artifact of hepatocyte cell death. We earlier demonstrated that the delayed activation of A2BAR with its agonist BAY 60–6583 provided significant protection against APAP-induced liver injury, as evidenced by reduced ALT levels and a decrease in the necrotic area [16]. We next examined whether or not this also influenced A2BAR mitochondrial translocation. Interestingly, delayed treatment with BAY 60–6583 could enhance the mitochondrial localization of A2BAR (Figure 3B), indicating that receptor activation modulates A2BAR trafficking.

### 3.2. Generation of Reactive Metabolite and JNK Activation Is Required for A2BAR Mitochondrial Translocation

During APAP hepatotoxicity, the generation of the reactive metabolite N-acetyl-p-benzoquinone imine (NAPQI), activation of c-Jun N-terminal kinase (JNK) and its mitochondrial translocation are critical steps required for hepatocyte necrosis [27]. Our previous studies have shown that 4-methylpyrazole (4MP) prevents the formation of NAPQI by inhibiting Cyp2E1 activity [20], and functions as a JNK inhibitor when administered 90 min after an APAP overdose [17] to prevent APAP-induced hepatocyte necrosis. To evaluate whether or not NAPQI formation and sustained JNK activation were necessary for the mitochondrial translocation of A2BAR, experiments were repeated with the administration of 4MP as a co-treatment with APAP (to inhibit Cyp2E1 and prevent the formation of NAPQI) or 90 min after APAP overdose (to allow initial NAPQI formation and JNK activation but prevent sustained JNK activation and the amplification of mitochondrial oxidant stress) [17,28,29]. The analysis of mitochondrial A2BAR localization 24 h after APAP revealed that both co-treatment and post-treatment with 4MP prevented A2BAR mitochondrial translocation (Figure 3C), indicating that the generation of NAPQI as well as the amplified mitochondrial oxidant stress due to sustained JNK activation was necessary for A2BAR’s movement to mitochondria.

### 3.3. A2BAR Is Translocated to the Outer Mitochondrial Membrane and Has a Similar Localization to That of PGRMC1

**Figure 4.**
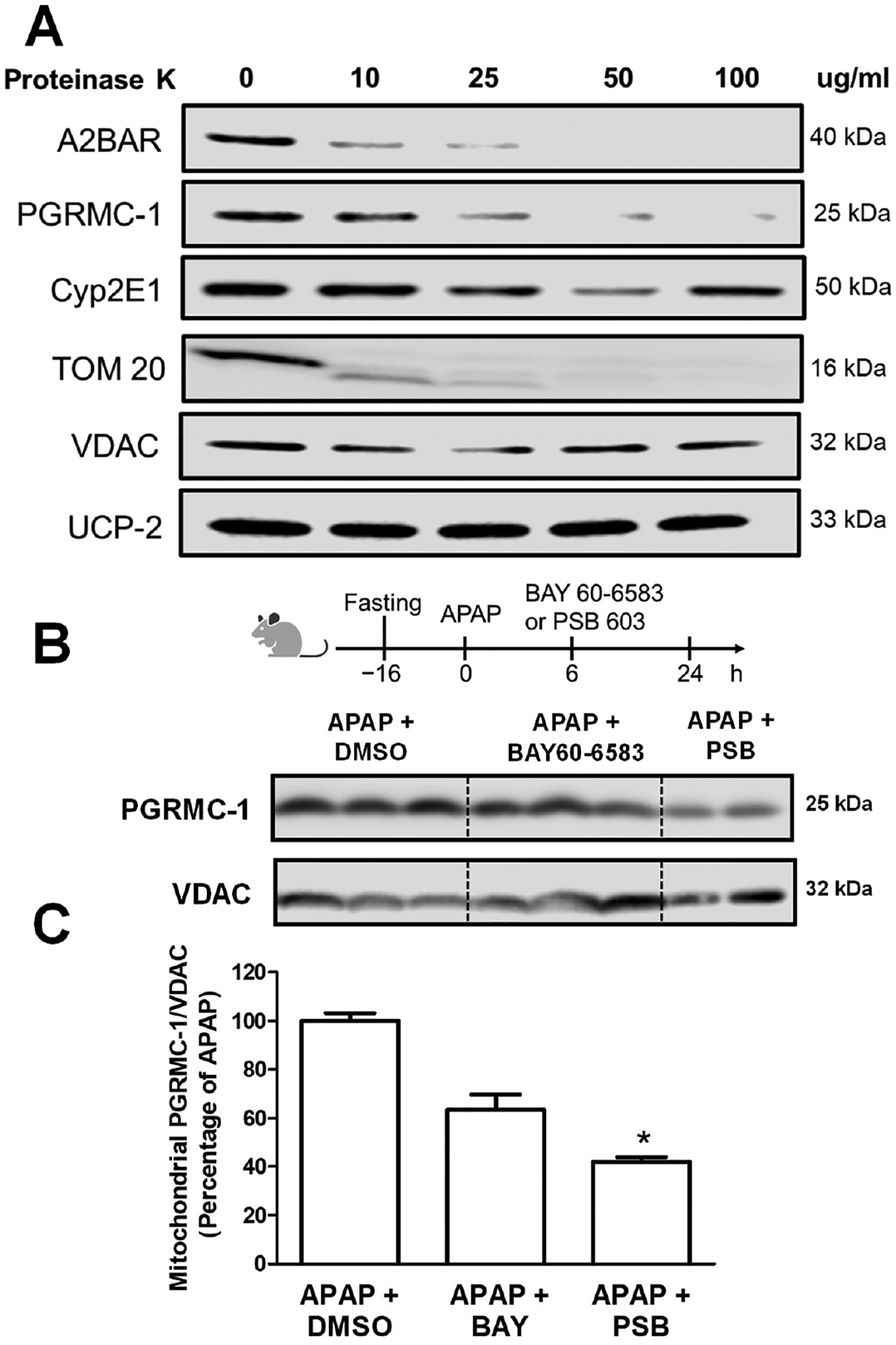
Putative A2BAR and PGRMC1 in the mitochondrial outer membrane. Mice were administered 300 mg/kg of APAP followed by BAY 60–6583 (4 mg/kg) or PSB 603 (1 mg/kg) 6 h later, and liver Livers tissue was collected 24 h after APAP overdose. (**A**)Western blot showing the susceptibility of mitochondrial A2BAR, PGRMC1, and CYP2E1 (24 h post-APAP) to digestion by increasing concentrations of proteinase K in comparison to other mitochondrial proteins. (**B**) PGRMC1 mitochondrial levels. (**C**) Densitometry of mitochondrial PGRMC1. * p < 0.05 (compared with APAP + DMSO group).

The next series of experiments evaluated A2BAR localization within the mitochondria in comparison to that of other relevant proteins such as PGRMC1 and CYP2E1. In order to investigate whether proteins were present on the outer or inner mitochondrial membranes, or the matrix, we performed the digestion of isolated mitochondria with progressively increasing concentrations of proteinase K, which would have selectively degraded accessible proteins on the outer mitochondrial membrane but not on the inner membrane. Hepatic mitochondria isolated 24 h after an APAP overdose were treated with proteinase K, and the integrity of A2BAR was compared to that of canonical mitochondrial membrane proteins. We used both TOM20 and VDAC as outer membrane protein controls, to determine their orientation on the outer membrane since TOM20 has a cytosolic domain, while VDAC is exclusively localized within the outer membrane, being a multi-pass membrane protein [30]. UCP-2 was used as a marker for the inner mitochondrial membrane. As seen in Figure 4A, A2BAR was sensitive to proteinase K digestion, similarly to TOM-20, while both VDAC and UCP-2 were resistant to proteinase K digestion. This demonstrates that A2BAR is anchored to the outer surface of the mitochondrial outer membrane. PGRMC1 showed a similar localization to A2BAR, indicating that it is also present on the mitochondrial outer membrane in mice after APAP overdose, while CYP2E1 was protected from proteinase K digestion, confirming its localization within the mitochondrial inner membrane [31].

### 3.4. A2BAR Possibly Binds to PGRMC1 on the Outer Mitochondria Membrane

**Figure 5.**
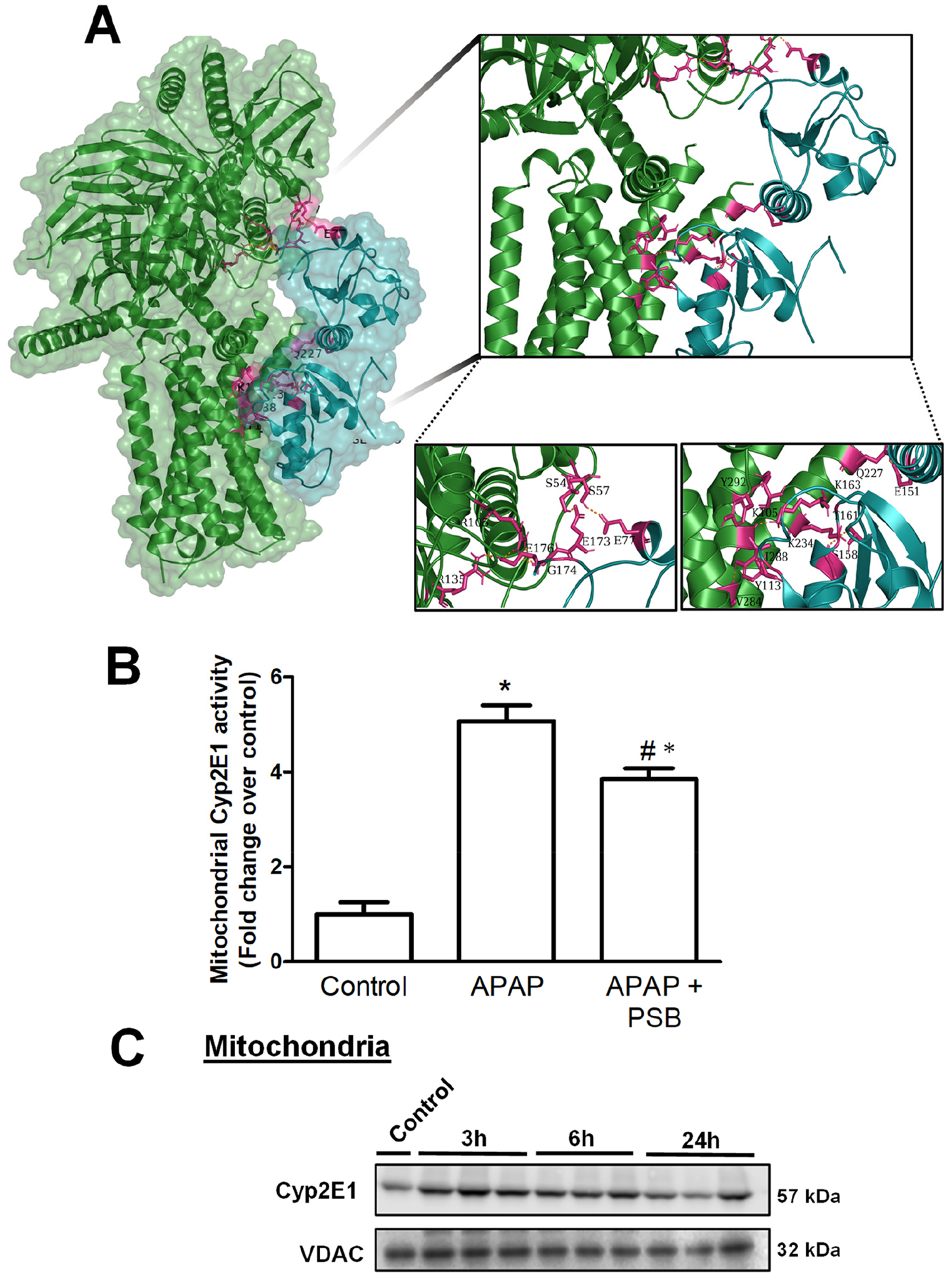
Putative A2BAR interaction with PGRMC1 and its influence on Cyp2E1 activity. (**A**) Surface representation of docked complex showing the interaction of A2BAR (green) with PGRMC1 (cyan) with a cleft formed by residues. Mice were administered a dose of 300 mg/kg of APAP, followed by treatment with PSB 603, 6 h later. After 24 h, liver tissue was collected, and the mitochondrial fraction was isolated. (**B**) Mitochondrial Cyp2E1 activity and (**C**) mitochondrial Cyp2E1 protein levels after APAP overdose. Data represent means ± SE of n = 3–4 animals per group. * p < 0.05 (compared with control), # p < 0.05 (compared to APAP).

Our localization of PGRMC1 to the outer mitochondrial membrane in liver mitochondria is relevant since the protein consists of an N-terminal region transmembrane domain and a C-terminal region with a cytochrome b5-like motif heme-binding domain, with independent proteinase K studies characterizing PGRMC1 as being associated with the outer face of the outer mitochondrial membrane [14]. We then examined PGRMC1 levels in mitochondria after APAP overdose and evaluated whether A2BAR agonists or antagonists could modulate mitochondrial PGRMC1. Animals were injected with BAY 60–6583 (A2BAR selective agonist) or PSB 603 (A2BAR selective antagonist) 6 h after receiving 300 mg/Kg of APAP, and PGRMC1 expression was evaluated 24 h post-APAP. While significant mitochondrial PGRMC1 was detectable after APAP, post-treatment with BAY 60–6583 showed a minor decrease that was not significant. Interestingly, the A2BAR antagonist PSB 603 showed a significant decrease in mitochondrial PGRMC1 (Figure 4B,C), suggesting that A2BAR influences PGRMC1 mitochondrial localization. To further examine the mechanism of A2BAR interaction with PGRMC1, we carried out molecular simulations using ClusPro [21]. Due to the lack of a complete crystal structure of PGRMC1, we performed this simulation using the PGRMC1 cytosolic domain (a.a.72–195) [32] and A2BAR coupled to a modified Gs protein [33]. ClusPro generates model structure-based pairwise interactions and energy functions to provide near-native models after docking. The size of each cluster represents the width of the corresponding energy and provides information on entropic contributions to the free energy [21]. For the interaction of A2BAR with the cytosolic domain of PGRMC1, the cluster that was most highly ranked using ClusPro criteria presented 81 members with a low energy of *−*1491.2. But how does this compare to known binding partners for PGRMC1? PGRMC1 has been shown to bind and regulate cytochrome P450 enzymes including Cyp2E1 [13]. To compare the binding energy obtained between A2BAR and PGRMC1 to that of its known binding partners, we next evaluated the interaction of the PGRMC1 cytosolic domain with Cyp2E1 (PDB 3E4E) [34] using ClusPro. For the interaction of PGRMC1 with Cyp2E1, the highest-ranked cluster had 104 members and an energy of *−*1016.7. This indicates that the energy of interaction between A2BAR and PGRMC1 is comparable to that of other known binding partners, suggesting that this interaction could be biologically relevant. Further analysis indicated that A2BAR–PGRMC1 binding is predominantly due to polar interactions among 18 residues (9 residues from A2BAR and 10 PGRMC1 residues) (Figure 5A). We further used scoring algorithms to characterize the docked structures, evaluating binding free energy, interfacial contact, and the free energy of the interface’s conformation using the PRODIGY webserver. The A2BAR–PGRMC1 complex shows a ΔG of *−*14.7 kcal mol^*−*1^ and a dissociation constant, Kd, of 1.6 × 10^*−*11^ M at 25 °C. This compares to the ΔG of 7.7 kcal mol–^1^ and the dissociation constant, Kd, of 2.2 × 10^*−*6^ M at 25 °C for the PGRMC1–Cyp2E1 complex. Taken together, all these data strongly suggest the possibility that A2BAR directly interacts with PGRMC1 and that this interaction is likely of biological relevance.

### 3.5. A2BAR–PGRMC1 Binding Influences Mitochondrial Cyp2E1 Activity after APAP Overdose

The data so far indicate that mitochondrial oxidative stress after an APAP overdose induces the translocation of A2BAR to the outer mitochondrial membrane where it interacts with PGRMC1. But what is the biological relevance of A2BAR interaction with PGRMC1 in mitochondria in the context of APAP-induced liver injury? While it is well recognized that Cyp2E1-mediated APAP metabolism to NAPQI is necessary for downstream events in APAP pathophysiology, the relevance of the subcellular localization of Cyp2E1 in this context is less understood. Interestingly, Cyp2E1 has been shown to be localized to liver mitochondria in addition to the endoplasmic reticulum [35] and PGRMC1 has been shown to be required for maximal Cyp1a2 and Cyp2e1 activity in the liver [13]. Thus, the presence of PGRMC1 on mitochondria could influence Cyp2E1 activity, and this was investigated using 7-ethoxy-4-trifluoromethyl coumarin (7-EFC), which is a Cyp2E1 substrate [36,37]. As seen in Figure 5B, APAP administration resulted in a significant 5-fold elevation in mitochondrial Cyp2E1 activity by 24 h, which was significantly inhibited by treatment with the A2BAR antagonist PSB 603. This increase in Cyp2E1 activity was not due to a substantial increase in Cyp2E1 protein levels in mitochondria since these were not significantly different with time after APAP overdose (Figure 5C).

## 4. Discussion

### 4.1. A2BAR Localization and Trafficking in Hepatocytes

Our earlier studies evaluating late-acting interventions against liver injury induced by an APAP overdose revealed that the neuronal guidance cue netrin-1 enhanced liver recovery and regeneration after an APAP overdose without influencing APAP-induced injury [15]. This effect was mediated through the adenosine A2B receptor, the delayed activation of which with the agonist BAY 60–6583 also facilitated liver recovery [16]. While A2BAR belongs to the family of G-protein-coupled receptors [1], it has a low affinity for adenosine [2], suggesting alternate functions for the receptor. In fact, A2BAR is now implicated in a wide range of pathophysiological conditions ranging from fibrosis to cancer [38]. We observed significant elevations in hepatic A2BAR mRNA and protein levels within hepatocytes after an APAP overdose [15]. Since A2BAR trafficking within cells is important for their physiological function [39] and there is limited information in this context within hepatocytes, we evaluated this in mice after APAP overdose. Our data indicate that A2BAR is mainly localized intracellularly as well as on the plasma membrane in hepatocytes at baseline. However, A2BAR translocation to mitochondria occurs within hours after APAP treatment, and experiments with 4MP intervention suggest that this trafficking is influenced by mitochondrial oxidant stress. This finding is not entirely surprising since A2BAR was also seen to be intracellular in freshly isolated human neutrophils [40] as well as in osteoclasts [41]. Mechanistically, this lack of exclusive plasma membrane localization could be because A2BAR does not have the dominant forward transport signal for export from the endoplasmic reticulum to the cell surface [42], which is also reflected in the poor expression of A2BAR at the cell surface in AD293 cells, a derivative of HEK 293 human embryonic kidney cells [42].

### 4.2. Mitochondrial Oxidant Stress and A2BAR Trafficking

The mitochondrial localization of A2BAR has been shown earlier in isolated cardiomyocytes [7] under baseline conditions, but this does not seem to be the case in hepatocytes where mitochondrial translocation only occurred after APAP exposure and the induction of mitochondrial oxidant stress. This would be similar to other cell types such as intestinal epithelial cells, where it was shown that the bulk of A2BAR is retained within the intracellular compartment during resting states [4], with a translocation to the plasma membrane only after stimulation [4]. Oxidative stress has been implicated in the trafficking of various receptors, and it was shown that exposure to hydrogen peroxide resulted in the rapid delivery of a cohort of human thromboxane receptors to the cell surface [43]. Oxidative stress can also influence the internalization and recycling of other GPCRs like the parathyroid hormone receptor [44], and tyrosine nitration was shown to impair the intracellular trafficking of FSHR to the cell surface [45]. It has also been demonstrated that NAD(P)H oxidase 4, a main source of reactive oxygen species in various cell types [46], can activate A2BAR on vascular smooth muscle cells [47]. This regulation can be reciprocal since A2BAR was also shown to inhibit superoxide production from mitochondrial complex I in rabbit cardiomyocytes [23] and regulate mitochondrial ROS production in neutrophils [48]. Mitochondrial oxidant stress plays a pivotal role in the progression of APAP-induced liver injury [49]. The activation of the cytosolic MAP kinase JNK and its subsequent translocation to the mitochondria significantly amplifies mitochondrial oxidative stress, precipitating the mitochondrial permeability transition (MPT) and the subsequent release of critical mitochondrial proteins, such as apoptosis-inducing factor (AIF) and endonuclease G (EndoG) [50]. Our prior investigations have demonstrated that the activation of A2BAR following APAP administration leads to a notable reduction in mitochondrial dysfunction, as evidenced by the diminished release of AIF and EndoG into the cytosol [16]. This suggests that the activation and translocation of A2BAR to the mitochondria in response to APAP overdose serves as a protective mechanism against APAP hepatotoxicity, effectively attenuating mitochondrial dysfunction and mitigating liver injury.

### 4.3. A2BAR Binding to PCRMC1 on Mitochondria

One of the critical proteins mediating APAP-induced liver injury that translocates to the mitochondria after an overdose is activated JNK, which does so to the outer mitochondrial membrane [51], and we find that A2BAR also localizes similarly after APAP. The novel finding, however, is the possible interaction of A2BAR with PGRMC1 on the mitochondrial outer membrane. PGRMC1 is a multifunctional cytochrome b5 (Cytb5) domain protein with important roles in membrane trafficking and mitochondrial function [52]. This A2BAR binding partner is even more important in the context of APAP pathophysiology since PGRMC1 binds and regulates cytochrome P450 enzymes in the endoplasmic reticulum [53,54] and cytochrome P450-mediated metabolism is critical for APAP-induced liver injury. While PGRMC1 may alternate between membrane-bound and cytosolic forms and has been identified in microsomal and mitochondrial fractions of rat adrenal inner zones [55], PGRMC1 was localized to the mitochondrial outer membrane in murine erythroleukemia (MEL) cells [14], similar to what we observed in hepatocyte mitochondria. Our molecular modeling data provide compelling evidence that A2BAR directly interacts with PGRMC1 and that the modulation of mitochondrial PGRMC1 levels by A2BAR antagonism further re-iterates this connection. Intriguingly, it was demonstrated that the nematode homologue of PGRMC1, VEM-1, physically interacts with C. elegans UNC-40, the nematode homologue of the mammalian cell surface receptor ‘deleted in colorectal cancer’ (DCC) [55], which is one of the receptors mediating netrin-1 function [56]. Since our earlier data showed no detectable DCC expression in livers after APAP [15], it is possible that A2BAR substitutes for DCC in the interaction with PGRMC1 in the context of APAP-induced liver injury in mammals.

### 4.4. A2BAR–PGRMC1 Binding Modulates CYP2E1 Activity

PGRMC1 has been shown to influence mitochondrial function by enhancing rates of mitochondrial respiration and fatty acid oxidation in a rat heart cardiomyocyte cell line [57] and also binds to and is required for maximal Cyp2e1 activity in the liver [53]. Cyp2E1 has been localized to liver mitochondria [12] and mitochondrial CYP2E1 has been suggested to be sufficient to mediate oxidative stress and cytotoxicity induced by ethanol and APAP [35]. While the electron donor used by mitochondrial CYP2E1 differs from that used by CYP2E1 in the endoplasmic reticulum [12], our data now suggest that PGRMC1 also controls mitochondrial CYP2E1 activity after APAP overdose. It is possible that PGRMC1 has additional roles in modulating mitochondrial function in the context of APAP pathophysiology since it has been shown that PGRMC1 reduces cardiac steatosis and lipotoxicity via the activation of fatty acid oxidation and mitochondrial respiration [57]. This is even more relevant since PGRMC1 knockout mice were shown to be protected against APAP-induced liver injury [13], suggesting that its modulation of mitochondrial Cyp2E1 activity and mitochondrial function in general could be critical to APAP-induced hepatocyte necrosis.

While this study provides valuable insights into the role of A2BAR trafficking in APAP-induced liver injury, several limitations warrant consideration. The study did not explore the long-term consequences of A2BAR localization to mitochondria or changes in localization during the recovery phase after APAP-induced liver injury. Regarding molecular modeling, it is important to note that utilizing a partial crystal structure of PGRMC1 to determine A2BAR–PGRMC1 interactions may affect ligand binding predictions, and the inability to capture post-translational modifications limits the understanding of functional states. Additionally, although the study identifies a link between A2BAR trafficking to mitochondria, its interaction with PGRMC1, and the modulation of mitochondrial Cyp2E1 activity, the broader mechanistic understanding of how these processes contribute to APAP-induced liver injury is not fully elucidated yet.

**Figure 6.**
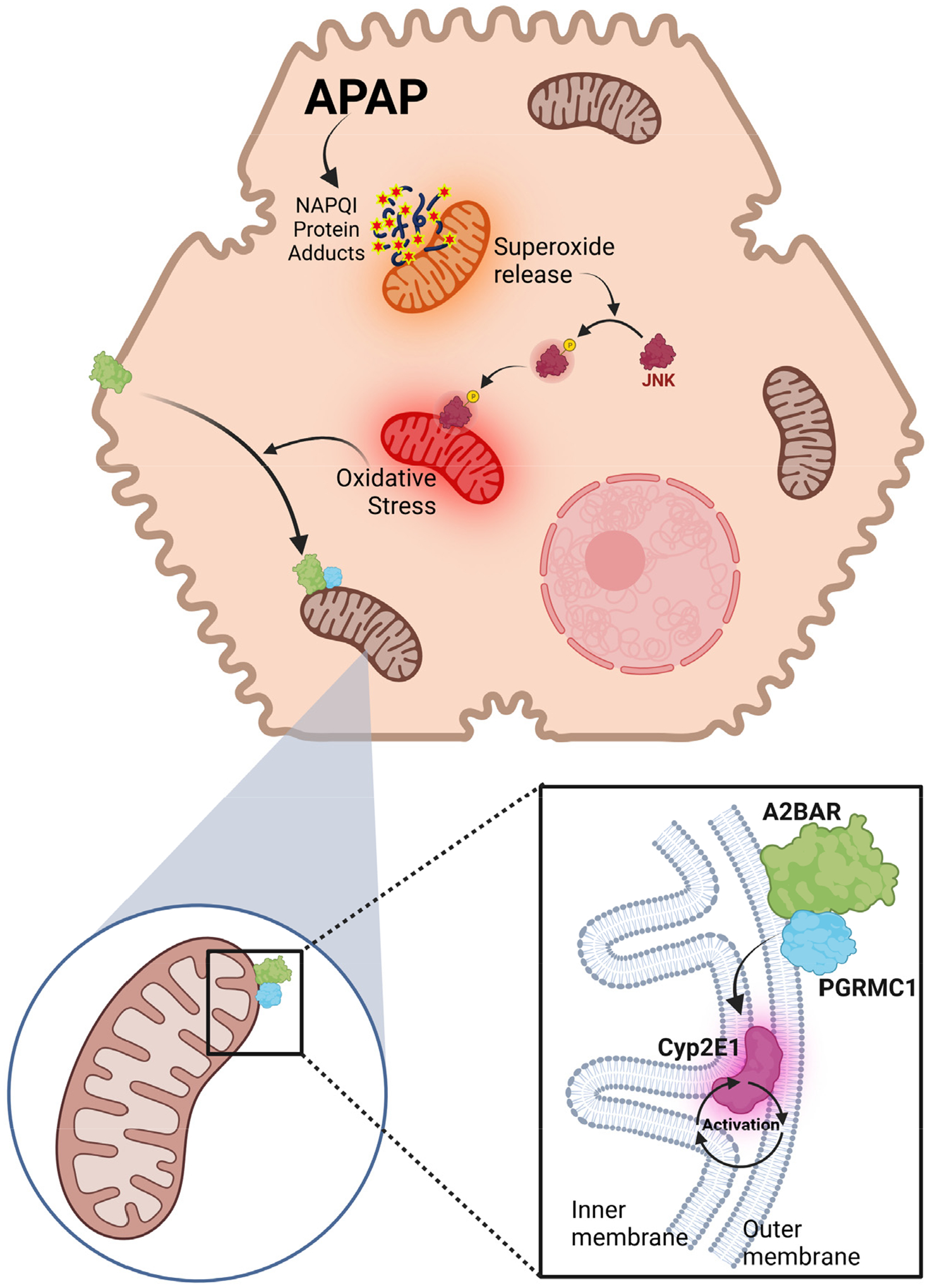
Diagram showing that PAP-induced mitochondrial oxidant stress causes A2BAR mitochondrial trafficking and the modulation of CYP2E1 activity through its binding to PCRMC1. Acetaminophen (APAP) overdose induces the formation of mitochondrial protein adducts, which induce superoxide release from the organelle into the cytosol. This activates the MAP kinase JNK, which translocates onto the mitochondria to amplify oxidative and nitrosative stress within the organelle. Our data indicate that this oxidative stress induces the translocation of the adenosine A2B receptor (A2BAR) from the plasma membrane onto the mitochondrial outer membrane, where it interacts with progesterone receptor component 1 (PGRMC1) to activate mitochondrial CYP2E1. Created with BioRender.com(accessed on 8 November 2023).

In conclusion, we have now identified a hitherto unknown consequence of mitochondrial oxidant stress after an APAP overdose, namely the activation of A2BAR trafficking to the organelle, which influences mitochondrial CYP2E1 activity. For the first time, to our knowledge, we localize A2BAR on the hepatic mitochondrial outer membrane after an APAP overdose and its interaction with the PGRMC1 (Figure 6). We also serendipitously uncovered a potential role for PGRMC1 in modulating mitochondrial Cyp2E1 activity in the context of APAP hepatotoxicity, which highlights the complexity of the organelle’s involvement in APAP pathophysiology. Further studies to unravel the specific roles of mitochondrial PGRMC1 in APAP-induced hepatocyte necrosis and its modulation by A2BAR are ongoing.

## Data Availability

Data is contained within the article.

## References

[1] Pasquini S; Contri C; Borea PA; Vincenzi F; Varani K Adenosine and Inflammation: Here, There and Everywhere. Int. J. Mol. Sci 2021, 22, 7685. 34299305 10.3390/ijms22147685 PMC8304851

[2] Beukers MW; den Dulk H; van Tilburg EW; Brouwer J; Ijzerman AP Why are A(2B) receptors low-affinity adenosine receptors? Mutation of Asn273 to Tyr increases affinity of human A(2B) receptor for 2-(1-Hexynyl)adenosine. Mol. Pharmacol 2000, 58, 1349–1356. 11093773 10.1124/mol.58.6.1349

[3] Aherne CM; Kewley EM; Eltzschig HK The resurgence of A2B adenosine receptor signaling. Biochim. Biophys. Acta 2011, 1808, 1329–1339. 20546702 10.1016/j.bbamem.2010.05.016 PMC2980804

[4] Sitaraman SV; Wang L; Wong M; Bruewer M; Hobert M; Yun CH; Merlin D; Madara JL The adenosine 2b receptor is recruited to the plasma membrane and associates with E3KARP and Ezrin upon agonist stimulation. J. Biol. Chem 2002, 277, 33188–33195. 12080047 10.1074/jbc.M202522200

[5] Sheth S; Brito R; Mukherjea D; Rybak LP; Ramkumar V Adenosine receptors: Expression, function and regulation. Int. J. Mol. Sci 2014, 15, 2024–2052. 24477263 10.3390/ijms15022024 PMC3958836

[6] Wang L; Kolachala V; Walia B; Balasubramanian S; Hall RA; Merlin D; Sitaraman SV Agonist-induced polarized trafficking and surface expression of the adenosine 2b receptor in intestinal epithelial cells: Role of SNARE proteins. Am. J. Physiol. Gastrointest. Liver Physiol 2004, 287, G1100–G1107. 15256361 10.1152/ajpgi.00164.2004

[7] Grube K; Rudebusch J; Xu Z; Bockenholt T; Methner C; Muller T; Cuello F; Zimmermann K; Yang X; Felix SB; Evidence for an intracellular localization of the adenosine A2B receptor in rat cardiomyocytes. Basic Res. Cardiol 2011, 106, 385–396. 21246204 10.1007/s00395-011-0151-6 PMC3533442

[8] Gnad T; Navarro G; Lahesmaa M; Reverte-Salisa L; Copperi F; Cordomi A; Naumann J; Hochhauser A; Haufs-Brusberg S; Wenzel D; Adenosine/A2B Receptor Signaling Ameliorates the Effects of Aging and Counteracts Obesity. Cell Metab 2020, 32, 56–70.e7. 32589947 10.1016/j.cmet.2020.06.006 PMC7437516

[9] Ramachandran A; Jaeschke H Mitochondria in Acetaminophen-Induced Liver Injury and Recovery: A Concise Review. Livers 2023, 3, 219–231. 37377765 10.3390/livers3020014 PMC10299745

[10] Lee SS; Buters JT; Pineau T; Fernandez-Salguero P; Gonzalez FJ Role of CYP2E1 in the hepatotoxicity of acetaminophen. J. Biol. Chem 1996, 271, 12063–12067. 8662637 10.1074/jbc.271.20.12063

[11] Cheung C; Yu AM; Ward JM; Krausz KW; Akiyama TE; Feigenbaum L; Gonzalez FJ The cyp2e1-humanized transgenic mouse: Role of cyp2e1 in acetaminophen hepatotoxicity. Drug Metab. Dispos 2005, 33, 449–457. 15576447 10.1124/dmd.104.002402

[12] Robin MA; Anandatheerthavarada HK; Fang JK; Cudic M; Otvos L; Avadhani NG Mitochondrial targeted cytochrome P450 2E1 (P450 MT5) contains an intact N terminus and requires mitochondrial specific electron transfer proteins for activity. J. Biol. Chem 2001, 276, 24680–24689. 11325963 10.1074/jbc.M100363200

[13] McGuire MR; Mukhopadhyay D; Myers SL; Mosher EP; Brookheart RT; Kammers K; Sehgal A; Selen ES; Wolfgang MJ; Bumpus NN; Progesterone receptor membrane component 1 (PGRMC1) binds and stabilizes cytochromes P450 through a heme-independent mechanism. J. Biol. Chem 2021, 297, 101316. 34678314 10.1016/j.jbc.2021.101316 PMC8591507

[14] Piel RB 3rd; Shiferaw MT; Vashisht AA; Marcero JR; Praissman JL; Phillips JD; Wohlschlegel JA; Medlock AE A Novel Role for Progesterone Receptor Membrane Component 1 (PGRMC1): A Partner and Regulator of Ferrochelatase. Biochemistry 2016, 55, 5204–5217. 27599036 10.1021/acs.biochem.6b00756 PMC5278647

[15] Duan L; Woolbright BL; Jaeschke H; Ramachandran A Late Protective Effect of Netrin-1 in the Murine Acetaminophen Hepatotoxicity Model. Toxicol. Sci 2020, 175, 168–181. 32207522 10.1093/toxsci/kfaa041 PMC7253199

[16] Duan L; Sanchez-Guerrero G; Jaeschke H; Ramachandran A Activation of the adenosine A2B receptor even beyond the therapeutic window of N-acetylcysteine accelerates liver recovery after an acetaminophen overdose. Food Chem. Toxicol 2022, 163, 112911. 35292334 10.1016/j.fct.2022.112911 PMC9018526

[17] Akakpo JY; Ramachandran A; Duan L; Schaich MA; Jaeschke MW; Freudenthal BD; Ding WX; Rumack BH; Jaeschke H Delayed Treatment with 4-Methylpyrazole Protects against Acetaminophen Hepatotoxicity in Mice by Inhibition of c-Jun n-Terminal Kinase. Toxicol. Sci 2019, 170, 57–68. 30903181 10.1093/toxsci/kfz077 PMC6592188

[18] Molck C; Ryall J; Failla LM; Coates JL; Pascussi JM; Heath JK; Stewart G; Hollande F The A(2b) adenosine receptor antagonist PSB-603 promotes oxidative phosphorylation and ROS production in colorectal cancer cells via adenosine receptor-independent mechanism. Cancer Lett 2016, 383, 135–143. 27693637 10.1016/j.canlet.2016.09.018

[19] Kotanska M; Szafarz M; Mika K; Dziubina A; Bednarski M; Muller CE; Sapa J; Kiec-Kononowicz K PSB 603—A known selective adenosine A2B receptor antagonist—Has anti-inflammatory activity in mice. Biomed. Pharmacother 2021, 135, 111164. 33385856 10.1016/j.biopha.2020.111164

[20] Akakpo JY; Ramachandran A; Kandel SE; Ni HM; Kumer SC; Rumack BH; Jaeschke H 4-Methylpyrazole protects against acetaminophen hepatotoxicity in mice and in primary human hepatocytes. Hum. Exp. Toxicol 2018, 37, 1310–1322. 29739258 10.1177/0960327118774902 PMC6482816

[21] Kozakov D; Hall DR; Xia B; Porter KA; Padhorny D; Yueh C; Beglov D; Vajda S The ClusPro web server for protein-protein docking. Nat. Protoc 2017, 12, 255–278. 28079879 10.1038/nprot.2016.169 PMC5540229

[22] Vangone A; Bonvin A PRODIGY: A Contact-based Predictor of Binding Affinity in Protein-protein Complexes. Bio Protoc 2017, 7, e2124. 10.21769/BioProtoc.2124 PMC8376549 34458447

[23] Yang X; Xin W; Yang XM; Kuno A; Rich TC; Cohen MV; Downey JM A2B adenosine receptors inhibit superoxide production from mitochondrial complex I in rabbit cardiomyocytes via a mechanism sensitive to Pertussis toxin. Br. J. Pharmacol 2011, 163, 995–1006. 21366548 10.1111/j.1476-5381.2011.01288.x PMC3130946

[24] Ramachandran A; Umbaugh DS; Jaeschke H Mitochondrial Dynamics in Drug-Induced Liver Injury. Livers 2021, 1, 102–115. 34485975 10.3390/livers1030010 PMC8412145

[25] Umbaugh DS; Nguyen NT; Jaeschke H; Ramachandran A Mitochondrial Membrane Potential Drives Early Change in Mitochondrial Morphology after Acetaminophen Exposure. Toxicol. Sci 2021, 180, 186–195. 33432343 10.1093/toxsci/kfaa188 PMC7916734

[26] Jiang Y; Dey S; Matsunami H Calreticulin: Roles in cell-surface protein expression. Membranes 2014, 4, 630–641. 25230046 10.3390/membranes4030630 PMC4194052

[27] Ramachandran A; Jaeschke H Acetaminophen hepatotoxicity: A mitochondrial perspective. Adv. Pharmacol 2019, 85, 195–219. 31307587 10.1016/bs.apha.2019.01.007 PMC7350903

[28] Nguyen NT; Du K; Akakpo JY; Umbaugh DS; Jaeschke H; Ramachandran A Mitochondrial protein adduct and superoxide generation are prerequisites for early activation of c-jun N-terminal kinase within the cytosol after an acetaminophen overdose in mice. Toxicol. Lett 2021, 338, 21–31. 33290831 10.1016/j.toxlet.2020.12.005 PMC7852579

[29] Du K; Ramachandran A; Jaeschke H Oxidative stress during acetaminophen hepatotoxicity: Sources, pathophysiological role and therapeutic potential. Redox Biol 2016, 10, 148–156. 27744120 10.1016/j.redox.2016.10.001 PMC5065645

[30] Walther DM; Rapaport D Biogenesis of mitochondrial outer membrane proteins. Biochim. Biophys. Acta 2009, 1793, 42–51. 18501716 10.1016/j.bbamcr.2008.04.013

[31] Sepuri NB; Yadav S; Anandatheerthavarada HK; Avadhani NG Mitochondrial targeting of intact CYP2B1 and CYP2E1 and N-terminal truncated CYP1A1 proteins in Saccharomyces cerevisiae--role of protein kinase A in the mitochondrial targeting of CYP2E1. FEBS J 2007, 274, 4615–4630. 17697118 10.1111/j.1742-4658.2007.05990.x

[32] Kabe Y; Nakane T; Koike I; Yamamoto T; Sugiura Y; Harada E; Sugase K; Shimamura T; Ohmura M; Muraoka K; Haem-dependent dimerization of PGRMC1/Sigma-2 receptor facilitates cancer proliferation and chemoresistance. Nat. Commun 2016, 7, 11030. 26988023 10.1038/ncomms11030 PMC4802085

[33] Cai H; Xu Y; Guo S; He X; Sun J; Li X; Li C; Yin W; Cheng X; Jiang H; Structures of adenosine receptor A(2B)R bound to endogenous and synthetic agonists. Cell Discov 2022, 8, 140. 36575181 10.1038/s41421-022-00503-1 PMC9794776

[34] Porubsky PR; Meneely KM; Scott EE Structures of human cytochrome P-450 2E1. Insights into the binding of inhibitors and both small molecular weight and fatty acid substrates. J. Biol. Chem 2008, 283, 33698–33707. 18818195 10.1074/jbc.M805999200 PMC2586265

[35] Knockaert L; Descatoire V; Vadrot N; Fromenty B; Robin MA Mitochondrial CYP2E1 is sufficient to mediate oxidative stress and cytotoxicity induced by ethanol and acetaminophen. Toxicol. In Vitro 2011, 25, 475–484. 21130154 10.1016/j.tiv.2010.11.019

[36] Buters JT; Schiller CD; Chou RC A highly sensitive tool for the assay of cytochrome P450 enzyme activity in rat, dog and man. Direct fluorescence monitoring of the deethylation of 7-ethoxy-4-trifluoromethylcoumarin. Biochem. Pharmacol 1993, 46, 1577–1584. 8240414 10.1016/0006-2952(93)90326-r

[37] Chang TK; Crespi CL; Waxman DJ Determination of CYP2B6 component of 7-ethoxy-4-trifluoromethylcoumarin O-deethylation activity in human liver microsomes. Methods Mol. Biol 2006, 320, 97–102. 16719378 10.1385/1-59259-998-2:97

[38] Vecchio EA; White PJ; May LT The adenosine A(2B) G protein-coupled receptor: Recent advances and therapeutic implications. Pharmacol. Ther 2019, 198, 20–33. 30677476 10.1016/j.pharmthera.2019.01.003

[39] Mundell S; Kelly E Adenosine receptor desensitization and trafficking. Biochim. Biophys. Acta 2011, 1808, 1319–1328. 20550943 10.1016/j.bbamem.2010.06.007

[40] Rosenberger P; Schwab JM; Mirakaj V; Masekowsky E; Mager A; Morote-Garcia JC; Unertl K; Eltzschig HK Hypoxia-inducible factor-dependent induction of netrin-1 dampens inflammation caused by hypoxia. Nat. Immunol 2009, 10, 195–202. 19122655 10.1038/ni.1683

[41] Knowles HJ The Adenosine A(2B) Receptor Drives Osteoclast-Mediated Bone Resorption in Hypoxic Microenvironments. Cells 2019, 8, 624. 31234425 10.3390/cells8060624 PMC6628620

[42] Moriyama K; Sitkovsky MV Adenosine A2A receptor is involved in cell surface expression of A2B receptor. J. Biol. Chem 2010, 285, 39271–39288. 20926384 10.1074/jbc.M109.098293 PMC2998132

[43] Ball SK; Field MC; Tippins JR Regulation of thromboxane receptor signaling at multiple levels by oxidative stress-induced stabilization, relocation and enhanced responsiveness. PLoS ONE 2010, 5, e12798. 20856817 10.1371/journal.pone.0012798 PMC2939892

[44] Ardura JA; Alonso V; Esbrit P; Friedman PA Oxidation inhibits PTH receptor signaling and trafficking. Biochem. Biophys. Res. Commun 2017, 482, 1019–1024. 27908723 10.1016/j.bbrc.2016.11.150 PMC5245921

[45] Zhou G; Hu RK; Xia GC; Yan SH; Ren QL; Zhao J; Wang FH; Huang CC; Yao Q; Tan Y; Tyrosine nitrations impaired intracellular trafficking of FSHR to the cell surface and FSH-induced Akt-FoxO3a signaling in human granulosa cells. Aging 2019, 11, 3094–3116. 31097679 10.18632/aging.101964 PMC6555443

[46] Weyemi U; Dupuy C The emerging role of ROS-generating NADPH oxidase NOX4 in DNA-damage responses. Mutat. Res 2012, 751, 77–81. 22580379 10.1016/j.mrrev.2012.04.002

[47] St. Hilaire C; Koupenova M; Carroll SH; Smith BD; Ravid K TNF-alpha upregulates the A2B adenosine receptor gene: The role of NAD(P)H oxidase 4. Biochem. Biophys. Res. Commun 2008, 375, 292–296. 18647598 10.1016/j.bbrc.2008.07.059 PMC2583397

[48] Herring SE; Mao S; Bhalla M; Tchalla EYI; Kramer JM; Bou Ghanem EN Mitochondrial ROS production by neutrophils is required for host antimicrobial function against *Streptococcus pneumoniae* and is controlled by A2B adenosine receptor signaling. PLoS Pathog 2022, 18, e1010700. 36374941 10.1371/journal.ppat.1010700 PMC9704767

[49] Jaeschke H; Duan L; Nguyen N; Ramachandran A Mitochondrial Damage and Biogenesis in Acetaminophen-induced Liver Injury. Liver Res 2019, 3, 150–156. 32655976 10.1016/j.livres.2019.10.002 PMC7351365

[50] Du K; McGill MR; Xie Y; Bajt ML; Jaeschke H Resveratrol prevents protein nitration and release of endonucleases from mitochondria during acetaminophen hepatotoxicity. Food Chem. Toxicol 2015, 81, 62–70. 25865938 10.1016/j.fct.2015.04.014 PMC4450137

[51] Win S; Than TA; Han D; Petrovic LM; Kaplowitz N c-Jun N-terminal kinase (JNK)-dependent acute liver injury from acetaminophen or tumor necrosis factor (TNF) requires mitochondrial Sab protein expression in mice. J. Biol. Chem 2011, 286, 35071–35078. 21844199 10.1074/jbc.M111.276089 PMC3186406

[52] Cahill MA Choose your partners for the next dance: Implied PGRMC1 roles in membrane trafficking and mitochondrial modulation. Fertil. Steril 2020, 113, 938–941. 32386619 10.1016/j.fertnstert.2020.01.029

[53] Hughes AL; Powell DW; Bard M; Eckstein J; Barbuch R; Link AJ; Espenshade PJ Dap1/PGRMC1 binds and regulates cytochrome P450 enzymes. Cell Metab 2007, 5, 143–149. 17276356 10.1016/j.cmet.2006.12.009

[54] Debose-Boyd RA A helping hand for cytochrome p450 enzymes. Cell Metab 2007, 5, 81–83. 17276348 10.1016/j.cmet.2007.01.007

[55] Cahill MA Progesterone receptor membrane component 1: An integrative review. J. Steroid Biochem. Mol. Biol 2007, 105, 16–36. 17583495 10.1016/j.jsbmb.2007.02.002

[56] Ranganathan P; Mohamed R; Jayakumar C; Ramesh G Guidance cue netrin-1 and the regulation of inflammation in acute and chronic kidney disease. Mediat. Inflamm 2014, 2014, 525891. 10.1155/2014/525891 PMC4065723 24991088

[57] Lee SR; Heo JH; Jo SL; Kim G; Kim SJ; Yoo HJ; Lee KP; Kwun HJ; Shin HJ; Baek IJ; Progesterone receptor membrane component 1 reduces cardiac steatosis and lipotoxicity via activation of fatty acid oxidation and mitochondrial respiration. Sci. Rep 2021, 11, 8781. 33888830 10.1038/s41598-021-88251-2 PMC8062525

